# CuGeO_3_ Nanoparticles: An Efficient Photothermal Theragnosis Agent for CT Imaging-Guided Photothermal Therapy of Cancers

**DOI:** 10.3389/fbioe.2020.590518

**Published:** 2020-11-19

**Authors:** Jiawu Wang, Chengyao Zhang

**Affiliations:** ^1^Department of Urology, The Second Affiliated Hospital of Chongqing Medical University, Chongqing, China; ^2^Department of Head and Neck Cancer Center, Chongqing University Cancer Hospital & Chongqing Cancer Institute & Chongqing Cancer Hospital, Chongqing, China

**Keywords:** photothermal theragnosis agent, multifunction, CT imaging, photothermal therapy, CuGeO_3_ nanoparticles

## Abstract

The photothermal agents have been widely developed due to the minimally invasive treatment for targeted tumor photothermal therapy, which is considered to have great potential for antitumor bioapplications. The development of multifunctional photothermal agents is extremely challenging. This work presents a novel photothermal theragnosis agent, i.e., CuGeO_3_ nanoparticles (CGO NPs), showing intense absorption in the near-infrared (NIR) window and excellent ability of CT imaging. Due to the strong NIR absorption, CGO NPs exhibit excellent photothermal effect with a photothermal conversion efficiency of 59.4%. Moreover, because of the high X-ray attenuation coefficient of germanium, the CGO NPs have a great potential of CT imaging diagnosis in clinical application. Additionally, the CGO NPs show negligible cytotoxicity *in vitro* and *in vivo*, indicating that it can be served as an outstanding contrast and anticancer agent in a biosafe way. Our work opens the way for the development of bimetallic copper-based oxides used in photothermal diagnostic agents for cancer treatment.

## Introduction

With the development of medical sciences, the average life expectancy of humans has been significantly improved, but cancer still remains a major threat. Early treatment of cancer, such as complete resection of the positive edge of the tumor, has a negative impact on health. Thus, the development of novel therapeutic methods is of great significance for improving cancer therapy (Lee et al., 2012; Liu et al., 2017). An ideal cancer treatment approach should be with low side effect, non-invasive, and targeting (Li and Pu, 2019). A large number of nanomaterials with low toxicity have been reported to efficiently promote photothermal therapy (PTT) for *in vivo* tumor ablation (Zhang et al., 2020). The interior properties of nanoparticles, including intrinsic optical and electrical properties, can be effective for PTT. The compounds of transition metal elements, which have a large atomic number and a variety of valences could attribute to strong absorption in the NIR region. Some of them also have a large X-ray attenuation coefficient, such as FeS, WO_x_, and Bi_2_S_3_ (Nam et al., 2019). Compared with metallic photothermal agents such as Au nanostructures (Lee et al., 2019), Ag nanoparticles, and Pt-based nanostructures (Huang et al., 2011), transition-metal nanoparticles possessing intense optical properties that are low cost, and have low cytotoxicity can serve as effective candidates for photothermal therapy of cancers (Chen et al., 2013).

The ternary semiconductor nanomaterials can inherit the performance of the corresponding binary semiconductors (Li et al., 2015, 2017; Wang et al., 2015). A series of studies have reported that ternary nanomaterials based on copper-based chalcogenide photosensitizers [for example, CuCo_2_S_4_ nanocrystals (Zhang et al., 2019), CuFeS_2_ nanoplates (Ding et al., 2017), or copper bismuth sulfide (Li et al., 2015)] could show several exciting properties such as strong absorption in the NIR region, excellent photothermal effect, and good photothermal stability. For example, copper–bismuth–sulfur nanostructures (such as Cu_3_BiS_3_) are usually p-type semiconductors. On the one hand, these nanomaterials may have many carrier concentrations due to copper defects and exhibit NIR absorption properties similar to binary copper-based chalcogenides (Li et al., 2015). On the other hand, the copper–bismuth–sulfur nanostructures can be used as efficient CT contrast agents like the Bi_2_S_3_ nanostructure due to the high X-ray attenuation coefficient of bismuth that has been extensively used for CT imaging (Ai et al., 2011). In theory, ternary copper-based semiconductor nanomaterials can show NIR absorption property and CT imaging ability. CuGeO_3_ nanoparticles have recently made some progresses in morphology and crystal phase control (Fu et al., 2018; O’Neal et al., 2018), but no CuGeO_3_ (CGO) nanoparticles with strong near-infrared absorption are synthesized (Bassi et al., 1996), which probably resulted from the low carrier concentration caused by monovalent copper ions (Tian et al., 2011). However, the concentration of the carriers in a semiconductor can be adjusted by doping and crystal defects (Li et al., 2014). These properties make the defect-structured CGO nanoparticles a new type of photothermal diagnostic agent.

In this work, CGO nanoparticles were designed and synthesized as biocompatible agents for photothermal therapy by a facile solvothermal method, which showed a favorable application in photothermal therapy (PTT) due to their small size, good dispersion, strong near-infrared (NIR) absorption, and excellent photothermal effect. In the CGO nanoparticles, the coexistence of different valences of copper ions attributes to the obvious absorption in the NIR region that resulted from the d-d energy band transition of Cu^2+^ (Tian et al., 2011; Li et al., 2015). As we expected, CGO nanoparticles showed an amazing photothermal effect with a photothermal conversion efficiency of 59.4%. CuGeO_3_ nanoparticles were first used as the photothermal agents for cancer treatment. When CT imaging was combined with photothermal therapy, CuGeO_3_ nanoparticles could enhance antitumor therapy due to the CT imaging-guided photothermal therapy. In addition, the good biocompatibility performance of Ge and Ge-containing compounds makes it possible to be used as dietary supplements (Gao et al., 2019; Song and Kazarian, 2020).

## Materials and Methods

### Synthesis of CGO Nanoparticles

CGO nanoparticles were synthesized via a simple solvothermal route. Normally, 0.7248 g of Cu(NO_3_)_2_⋅3H_2_O and 0.3139 g of GeO_2_ was fully dissolved in dehydrated ethanol (35 ml) with magnetic stirring at room temperature until the mixture solution completely dissolved. After 30 min of stirring, the NaOH solution (2 M, 100 μl) should be gently added into the ethanol dispersion of Cu(NO_3_)_2_ and GeO_2_ drop by drop. In this period, the magnetic stirrer should remain stirring for at least 20 min to make sure of a sufficient reaction. Next, the homogeneous solution was transferred into a Teflon-lined autoclave (50 ml in volume). The temperature of the electric oven should be 140°C, and the whole reaction may take 24 h. After the reaction, the Teflon-lined autoclave should cool down to room temperature, and the products were obtained by centrifuge and washing with deionized water. Finally, the as-prepared CGO nanoparticles were redispersed for further modification. In order to improve the compatibility of CGO NPs, 1 mg of PVP was added to the obtained CGO NP solution with a concentration of 1.25 mg/ml as a mixture solution. Similar with previous processes, the resulting solution should be centrifuged and washed twice with ethanol.

### Characterization

The transmission electron microscope (TEM) was used to determine the morphology, size, and microstructure of CGO nanoparticles. The UV-vis absorbance performance was determined by a UV-visible-NIR spectrophotometer at room temperature. The crystal structure of the samples was obtained by X-ray powder diffraction (XRD). X-ray photoelectron spectroscopy (XPS) was used for element composition and chemical state analysis in CGO nanoparticles. The concentration of germanium ions was determined by inductively coupled plasma atomic emission spectroscopy (ICP-AES). NIR lasers (808 nm) and an infrared thermal imager were used to obtain the infrared thermal images of dispersions to evaluate the photothermal effects of CGO nanoparticles. The power density of the infrared thermal imager could be adjusted from 0 to 2 W/cm^2^. The handheld optical power meter was used to calibrate the output power of the lasers independently.

### Photothermal Effect

The photothermal performance of the CGO nanoparticles has been measured by laser irradiation experiment. The gradient concentrations of CGO nanoparticle aqueous dispersion were irradiated under an 808 nm laser (0.5 W cm^–2^) for 5 min. The infrared thermal camera was used to monitor and record the temperature change in CGO nanoparticle dispersion.

In order to calculate the photothermal conversion efficiency (η) of CGO nanoparticles, we dispersed the CGO nanoparticles into deionized water, which was exposed to an 808 nm NIR laser (0.5 W cm^–2^, 5 min). The temperature curve during laser on and off was recorded. The photothermal conversion efficiency can be calculated by referring to the following equations:

(1) $$\eta =\frac{hA\left(\Delta T_{\max}-\Delta T_{\max,\text{water}}\right)}{I\left(1-10^{-A_{808}}\right)}$$

(2) $$\tau_{s}=\frac{m_{D}c_{D}}{hA}$$

where *I* is the NIR laser power, and *A*_808_ stands for the absorbance of dispersion at 808 nm. The value of *hA* can be calculated from Eq. 2 by using the system time constant *τ_s_* with the help of the mass (*m*_D_) and the heat capacity (*C*_D_) of deionized water. Δ*T*_*max*_ and Δ*T*_*max*,water_ are the temperature change in CGO nanoparticle dispersion and pure water.

### Cell Culture

The Cell Counting Kit-8 (CCK-8) has been used to evaluate the *in vitro* cytotoxicity of the CGO nanoparticles. CAL-27 cells were seeded into 96-well culture plates with a density of 1.5 × 10^4^ cells per well at 37°C with the presence of 5% CO_2_ for 24 h. Then, the various concentrations of the CGO nanoparticles (0–320 ppm) was added to each well and incubated for 24 h. Next, 10 μl of CCK-8 solution was added to each well of the plate for another 2 h incubation. Each sample was measured three times to reduce the sampling error.

### Photothermal Therapy *in vivo*

CAL-27 tumor-bearing nude mice were randomly divided into three groups: (a) mice with no treatments (Control); (b) mice injected with PBS injection and then irradiated with 808 nm laser irradiation with power density of 0.5 W/cm^2^ (PPS + NIR); (c) mice injected with CGO nanoparticle and then irradiated with 808 nm laser irradiation with power density of 0.5 W/cm^2^ (CGO + NIR). After the indicated treatment, the tumor size (∼100 mm^3^ before treatment) and body weights were measured every 2 days for 2 weeks. The calculation of the tumor volume and the relative tumor volume was determined by V/V_0_ (Gao et al., 2019).

### CT Imaging

The micro-CT imaging system was used to obtain the CT images of the various concentrations of CGO nanoparticles (0, 0.5, 1.0, 2.0, and 4.0 mg/ml). First, the prepared solution of CGO nanoparticles was directly scanned using a micro-CT imaging system to measure the CT signal of resulting products at 100 kV. The manufacturer-supplied software was used to evaluate the CT values on the same workstation.

All animal experiments were carried out in accordance with the guidelines of the Institutional Animal Care and Use Committee of the Second Affiliated Hospital of Chongqing Medical University. CAL-27 tumor-bearing mice were also divided into two groups: a. intratumoral injection of PBS (100 μl) and b. intratumoral injection of the CGO nanoparticles (4 mg/ml, 100 μl), and the mice were anesthetized before CT scanning. Thirty minutes after injection, CT scanning was performed with parameters similar to those for *in vitro* experiments.

### Long-Term Toxicity *in vivo*

H&E analysis was conducted to evaluate the effect of CGO on the main organs (including the heart, liver, lungs, spleen, and kidneys) after the intravenous administration of CGO nanoparticles. The contents of nanocrystals accumulated in the main organs at different time points (i.e., 1, 5, 10, 15 days) were also evaluated to study the biodistribution.

## Results and Discussion

**FIGURE 1:**
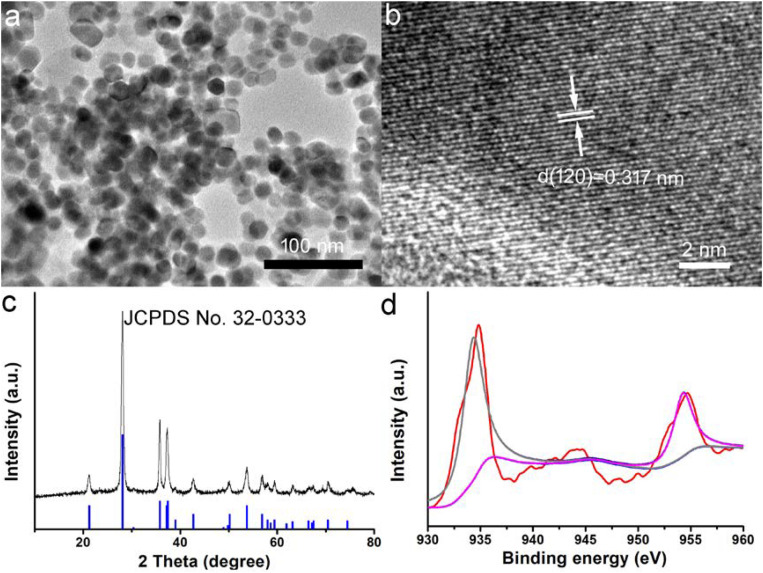
**(a)** Transmission electron microscope (TEM) image of as-prepared CuGeO_3_ nanoparticles (CGO NPs). **(b)** CGO NP microstructure under a high-resolution TEM. **(c)** X-ray powder diffraction (XRD) patterns of synthesized CGO and standard CGO on a JCPDS card (no. 32-0333). **(d)** X-ray photoelectron spectroscopy (XPS) spectrum for copper ion in the CGO nanoparticles.

CGO nanoparticles were synthesized by a simple solvothermal method and subsequently modified the surface by PVP. The transmission electron microscopy (TEM) image of CGO nanoparticles is shown in Figure 1a. It exhibits that the resulting products have an irregular morphology and dimensions of around 25 nm (Supplementary Figure S1). Obviously, the as-prepared CGO nanoparticles was non-aggregated in the TEM image (Figure 1a), showing very good dispersion. The small size of CGO nanoparticles can be considered perfect for photothermal agents as the nanoparticles with this size could have relatively long blood circulation. More microstructure information is shown in Figure 1b from the high-resolution TEM (HRTEM). The lattice spacing from the HRTEM was determined to be 0.317 nm, assigned to (120) planes of orthorhombic structured CuGeO_3_. The XRD analysis of the CGO products shown in Figure 1c revealed that the obtained products have a high crystallinity for pure CGO because all of the main reflection peaks of CGO nanoparticles can be matched well with the standard cards JCPDS file no. 32-0333, indicating the formation of the orthorhombic structured of CuGeO_3_. In Figure 1d, a high resolution of XPS spectrum for Cu 2p in the CGO nanoparticles was analyzed to verify the existence of non-equivalent valency state of copper ions in CGO NPs. There are three sharp peaks that can be observed in Figure 1d. The peaks at 932.7 and 952.6 eV indicated the presence of Cu^+^ in as-prepared CuGeO_3_ nanoparticles; the peaks at 935.1, 941.6, and 955.2 eV belonged to Cu^2+^ in CuGeO_3_ nanoparticles (Lv et al., 2016; Suriyawong et al., 2016). The XPS spectrum demonstrated that there was a coexistence of copper ions with different valences in synthesized CuGeO_3_ nanoparticles, probably resulting in intense NIR absorption due to d-d electron transition. As reported before, the existence of different valency ions in transition metal-based photothermal agents could result in ionized free carriers, thus contributing to NIR absorption (Li et al., 2014).

**FIGURE 2:**
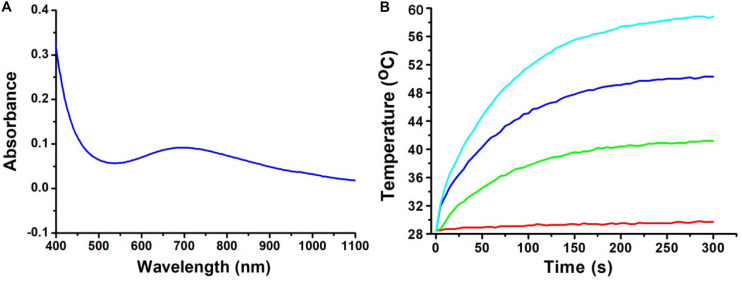
**(A)** UV-vis absorption spectrum for CuGeO_3_ NPs with a wide range of 400–1,000 nm. **(B)** Temperature elevation of the CuGeO_3_ nanoparticle solution with different concentrations under irradiation with an 808 nm laser (0.5 W/cm^2^, 300 s).

The optical properties of the CGO nanoparticles were investigated by a UV-vis-NIR spectrophotometer in a range from 400 to 1,000 nm. The spectrum of the CGO nanoparticle aqueous dispersion displayed high absorption in both the visible and near-infrared regions, as shown in Figure 2A. The absorbance value was high enough in the NIR region from 600 to 1,100 nm, which showed the typical absorption properties of ternary copper-based compounds (Li et al., 2015; Yuan et al., 2020). By determining the concentration of CGO nanoparticles via ICP-AES, the extinction coefficient of the CGO nanoparticles at 808 nm was measured to be 11.3 L g^–1^ cm^–1^. We then investigated the photothermal performance of CuGeO_3_ NPs. The as-prepared CGO NPs with gradient concentrations of 0, 20, 40, 80 ppm were irradiated by an 808 nm laser (0.5 W cm^–2^) for 5 min. In Figure 2B, we can see that the temperature change showed an obvious difference between the CGO nanoparticle aqueous dispersion and pure water in the photothermal effect experiment. The temperature can easily reach to ∼55°C in 5 min, which was high enough to kill tumor cells for CGO nanoparticle aqueous solution with low concentration (80 ppm). However, the temperature of pure water, as a control, remained almost unchanged (less than 2°C). According to a previous report, the reproduction and growth of tumor cells can be suppressed at temperatures of 40–44°C because a high temperature can result in DNA damage, protein denaturation, and disruption of the cellular membrane, leading to eradication of tumor tissues (Nam et al., 2019).

**FIGURE 3:**
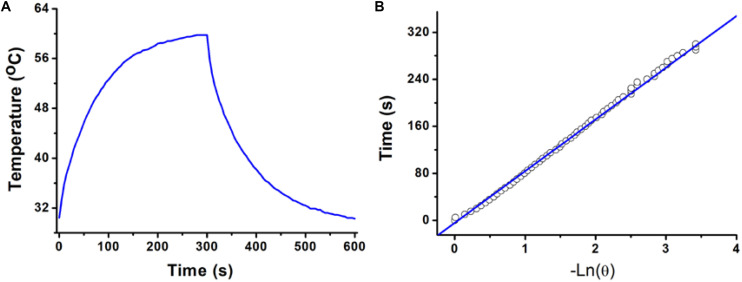
**(A)** Photothermal effect of a CuGeO_3_ NP aqueous solution (50 ppm) irradiated under an 808 nm laser with a power density of 0.5 W/cm^2^, in which the process lasted for 5 min, and the laser was then shut off after irradiation. **(B)** Time constant for heat transfer from the system, determined to be τ_s_ = 93.1 s.

The photothermal conversion efficiency of copper-based agents has great influences on their photothermal performance. To further explore the optical performance of the CGO nanoparticles, the photothermal conversion efficiency was measured following Tian’s report (Tian et al., 2013). With the continuous irradiation of the 808 nm laser (0.5 W/cm^2^, 300 s), the temperature change in the solution (80 ppm) was recorded until the temperature reached a steady-state condition (Figures 3A,B). Obviously, temperature elevation of the solution (50 ppm) reached to 51.3°C (the highest point) under irradiation for 5 min. The temperature of the solution was decreased to room temperature with time extending after the laser was shut off. Based on the obtained data, the heat conversion efficiency, η, of CuGeO_3_ NPs driven by an 808 nm laser can reach to about 59.4%. Thus, we supposed that the high photothermal conversion efficiency of these nanoparticles resulted from intense absorbance in the NIR region, which makes it possible for photothermal therapy of cancers.

**FIGURE 4:**
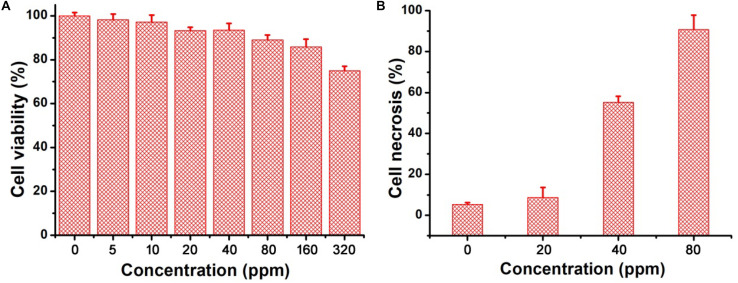
**(A)** *In vitro* cytotoxicity of CGO nanoparticles on CAL-27 cells. **(B)** Relative cell necrosis of CAL-27 cells after an 808 nm laser irradiating with different concentrations of CGO nanoparticles.

The excellent photothermal performance of CGO nanoparticles motivated us to further evaluate the potential of these nanoparticles *in vivo*. Prior to the bioapplication, the cytotoxicity *in vitro* of CGO nanoparticles on cells was carried out by a CCK-8 assay. The cell viability remained at a high level (over 85% when the concentration was increased to 160 ppm) after incubation for 24 h (Figure 4A), indicating the excellent biocompatibility of CGO nanoparticles. Furthermore, the photothermal therapy effect *in vitro* on CAL-27 cells was evaluated by the combination of CGO nanoparticles and 808 nm lasers. The cell viability after the treatment was investigated by a standard CCK-8 evaluation. Lasers (808 nm) with a power density of 0.5 W cm^–2^ were used to irradiate CAL-27 cells with varied concentrations of CGO nanoparticles. As shown in Figure 4B, CGO nanoparticles (80 ppm) that mediated NIR irradiation could induce over 85% cancer cell death at a low power density of irradiation for 10 min (0.8 W/cm^2^), indicating the excellent photothermal therapy effect *in vitro*.

**FIGURE 5:**
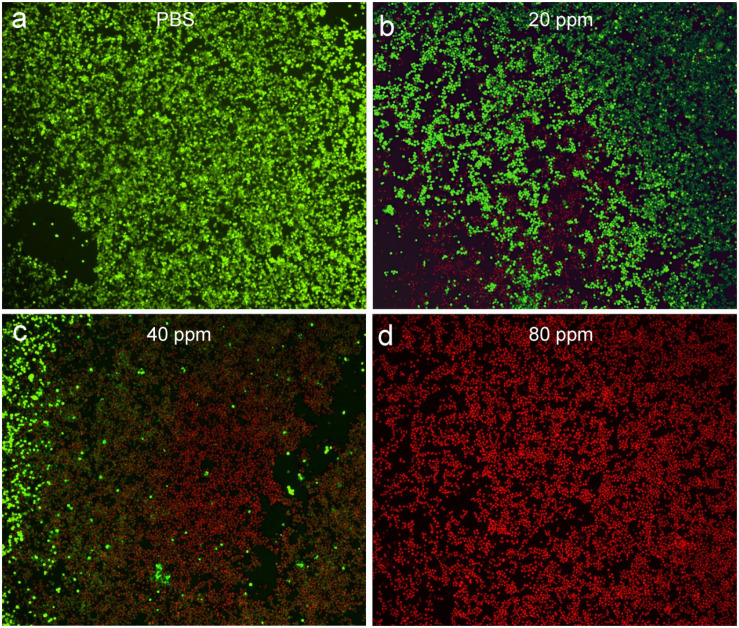
Confocal microscopy images of CAL-27 cells after incubation with various concentrations of CGO nanoparticles for phototherapy: **(a)** PBS, **(b)** 20 ppm, **(c)** 40 ppm, and **(d)** 80 ppm. The CAL-27 cells were costained with propidium iodide (red, dead cells) and calcein-AM (green, live cells). Magnification: 200×.

After costaining with propidium iodide and calcein-AM, the live/dead staining cells can be obviously observed under the confocal microscopy, which can help to evaluate photothermal effect *in vitro* using CGO nanoparticles due to the distinct chromatic aberration between live (green fluorescence) and dead (red fluorescence) staining cells. PBS buffer can be used as a control experiment. As can be seen in Figure 5a, only live cell staining (green) was present in the untreated cells. With the increasing concentration of CGO nanoparticle dispersion, massive cell death occurred as expected (Figures 5b–d). The cell mortality rate after the treatment could reach to ∼90% (Figure 5d), when CGO nanoparticles (80 ppm) was combined with the irradiation of an 808 nm laser, further certifying the efficient photothermal effect *in vitro*.

**FIGURE 6:**
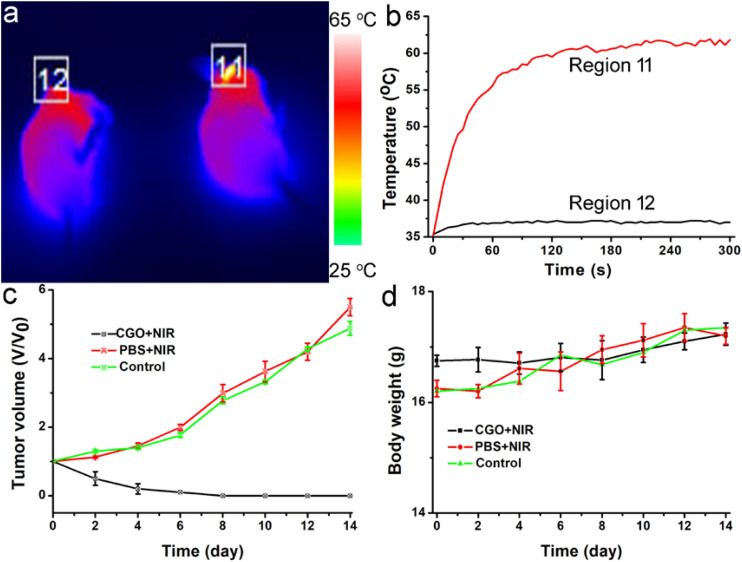
**(a)** Infrared thermal imaging pictures of mice treated with PBS (left) and CGO nanoparticles (right), then irradiated by 808 nm lasers with a power density of 0.5 W cm^–2^ for 300 s. **(b)** The temperature change curve of a local tumor with different treatments for 5 min. **(c)** Tumor growth curves in each group of mice after treatments. **(d)** Body weight changes in the mice in each group after the treatments.

Photothermal therapy *in vivo* using CGO nanoparticles was then carried out. The mice were randomly divided into three groups: (a) Control group; (b) PBS + 808 nm laser irradiation with power density of 0.5 W/cm^2^ (PBS + NIR); (c) CGO nanoparticle injection + 808 nm laser irradiation (CGO + NIR). The temperature change in mice was monitored with a thermal imaging camera during the whole treatment. As expected, the temperature raised rapidly in the experiment group (a), in sharp contrast to group (b) without injection of CGO. As shown in Figure 6a, the tumor site in the experiment group (injected with CGO nanoparticles) was much brighter than that of the control group (injected with PBS). That is to say that only mice injected with CGO nanoparticles can contribute to the above fantabulous photothermal effects *in vivo*. Tumor surface temperature curves (Figure 6b) revealed that the surface temperature of mice injected with CGO nanoparticles could dramatically increase to about 60°C with the role of an 808 nm laser, while the surface temperature of mice injected with CGO nanoparticles was still kept at room temperature. Then, the relative tumor volumes have been recorded to evaluate the antitumor efficiency for 14 days (Figure 6c). The tumor growth significantly disappeared in the mice of group (c) sharply compared to the two other control groups. Evidently, there was no reoccurrence observed in group (c) after 14 days of treatment, which means that the tumor cells of mice have disappeared completely, while continuous tumor development was found in the control groups. The difference in tumor changes after the treatments at the 14th day after treatments (Supplementary Figure S2) was matched well with the tumor growth curves in Figure 6c. After the treatment, the tumors from the three groups were also analyzed by H&E staining analysis (Supplementary Figure S3). Tumor cells in the CGO + NIR group were significantly damaged (nuclear shrinkage and nuclear dissolution), while tumor cells in the control group and the PBS + NIR group showed almost no changes. In addition, the side effect of the photothermal therapy could be evaluated by measuring the body weight change in the mice after the treatment. Figure 6d displayed that the body weight among the three groups of mice did not show notable differences, suggesting the limited biotoxicity of CGO at the given conditions.

**FIGURE 7:**
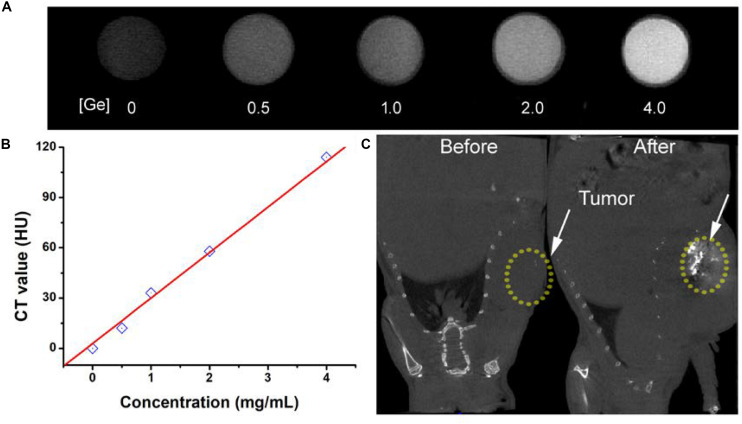
**(A)** *In vitro* CT images of CGO nanoparticles with different concentrations. **(B)** CT values as a function of the concentrations of CGO nanoparticles. **(C)** CT images *in vivo* of mice before and after the injection of CGO nanoparticles.

The early diagnosis of cancer remains a big challenge for antitumors. CT imaging, as a powerful diagnostic tool, has been widely used in clinics. It has been reported that a great deal of metallic elements have been developed as promising CT contrast agents, such as iodine, bismuth, lanthanides, gold, and so on. Germanium, as a typical metallic element, has a relatively high attenuation coefficient; thus, we believe that CGO nanoparticles also have great potential to become a contrast agent for CT imaging, so we evaluated the contrast efficacy of CGO nanoparticles. As displayed in Figures 7A,B, with the increase in Ge content, the CT signal enhanced fast (dark to light), and the signal intensity (Hounsfield units, HU) exhibited linear growth behavior. These results demonstrated that CGO nanoparticles could be an efficient CT contrast agent for cancer diagnosis. All the advantages above motivated us to further investigate the CT imaging diagnosis application of CGO nanoparticles *in vitro*. As depicted in Figure 7C, first, before intratumoral injection of CGO nanoparticles, the tumor appeared as a relatively dark image. After intratumoral injection, the tumor turned to full brightness at 2 h, demonstrating that CGO can be effective for imaging diagnosis.

Finally, we evaluated the long-term toxicity *in vivo* of CGO nanoparticles. After the intravenous administration of CGO nanoparticles, H&E analysis was conducted to evaluate the effect of CGO on the main organs. From the H&E staining of the major organs (including the heart, liver, lungs, spleen, and kidneys), no changes in the size, shape, and number of cells were observed (Supplementary Figure S4). The contents of nanocrystals accumulated in main organs at different time points were also evaluated. It showed (Supplementary Figure S5) that the CGO nanoparticles mainly accumulated at the kidneys and the spleen, indicating that CGO nanoparticles could be eliminated in the body through the kidneys and the spleen.

## Conclusion

In summary, CuGeO_3_ nanoparticles have great potential to be a novel bifunctional photothermal agent due to their small size (∼25 nm), good compatibility and dispersion, excellent photothermal effect, and CT imaging capability, which can be easily prepared by a simple solvothermal process and, subsequently, a surface modification process. The CuGeO_3_ nanoparticle solution reveals an intense and strong absorption band in the NIR region due to the existence of different valence states of copper ions and the excellent photothermal effect. The photothermal conversion efficiency of CuGeO_3_ nanoparticles was calculated to be 59.4%. Moreover, CuGeO_3_ nanoparticles can be used as CT contrast agents for CT imaging both *in vitro* and *in vivo* due to the high attenuation coefficient of germanium. Therefore, the CuGeO_3_ nanoparticles have great potential as a bifunctional agent with both high photothermal efficiency and CT imaging.

## Data Availability Statement

All datasets presented in this study are included in the article/Supplementary Material.

## Ethics Statement

The animal study was reviewed and approved by the Second Affiliated Hospital of Chongqing Medical University.

## Author Contributions

JW and CZ designed the project and wrote the manuscript. JW carried out the experiment and performed the experimental data analysis. Both authors contributed to discussion of the results.

## Conflict of Interest

The authors declare that the research was conducted in the absence of any commercial or financial relationships that could be construed as a potential conflict of interest.
