# Supplementary material for: CuGeO3 Nanoparticles: An Efficient Photothermal Theragnosis Agent for CT Imaging-Guided Photothermal Therapy of Cancers
Source: Front Bioeng Biotechnol. 2020 Nov 19;8:590518. doi: 10.3389/fbioe.2020.590518 (PMC7717952; doi:10.3389/fbioe.2020.590518)
Supplement: Supplementary file 1 [file Data_Sheet_1.docx]

Supporting Information

**CuGeO_3_ nanoparticles: an efficient photothermal theragnosis agent for CT imaging-guided photothermal therapy of cancers**

Jiawu Wang^1^, Chengyao zhang^2^*

^1^Department of Urology, the Second Affiliated Hospital of Chongqing Medical University, Chongqing 400010, China.

^2^Department of Head and Neck Cancer Center, Chongqing University Cancer Hospital & Chongqing Cancer Institute & Chongqing Cancer Hospital, Chongqing 400010, China.

^*^Email: CY. Zhang, cczhangcy@163.com.


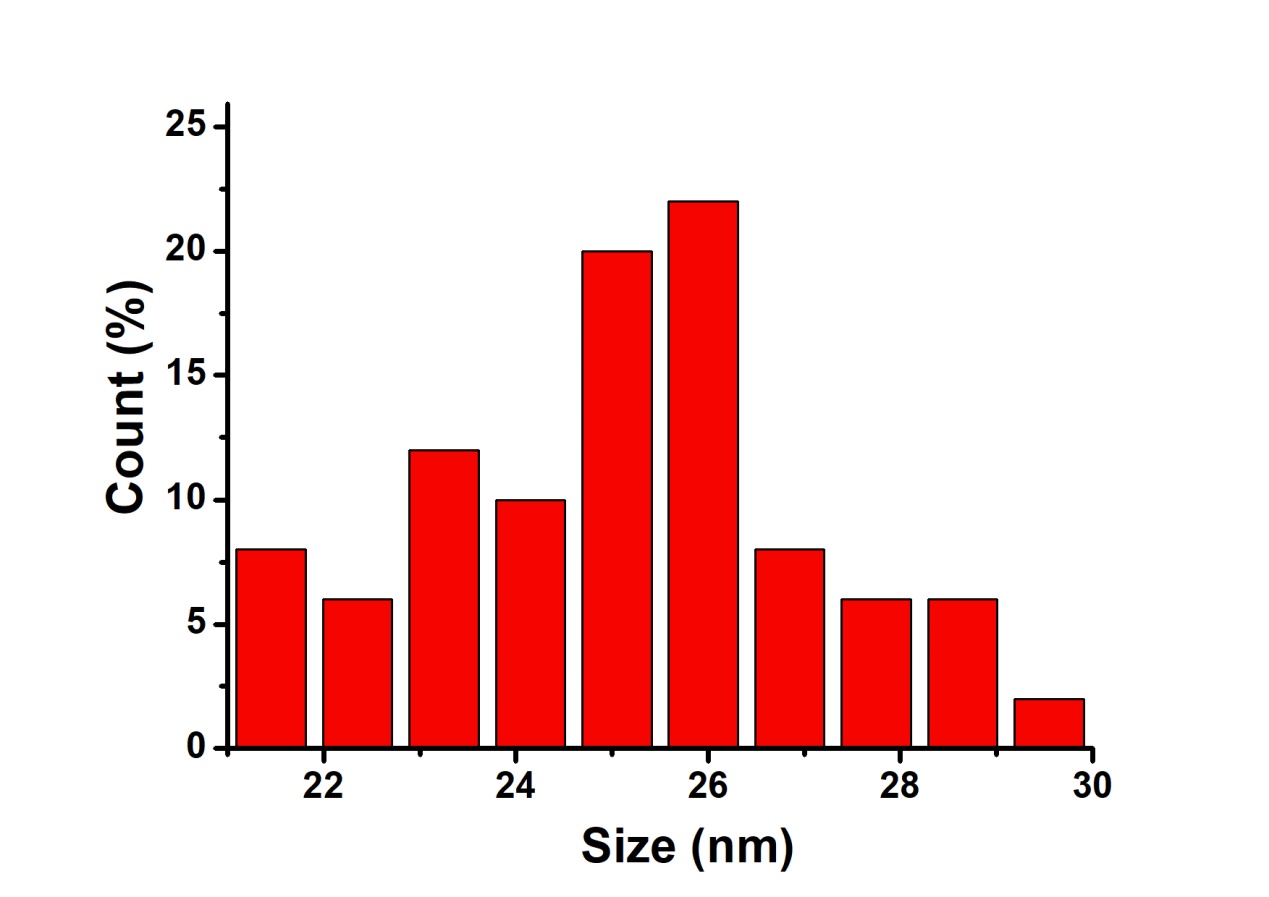


Figure S1. Size distribution of CGO nanoparticles.


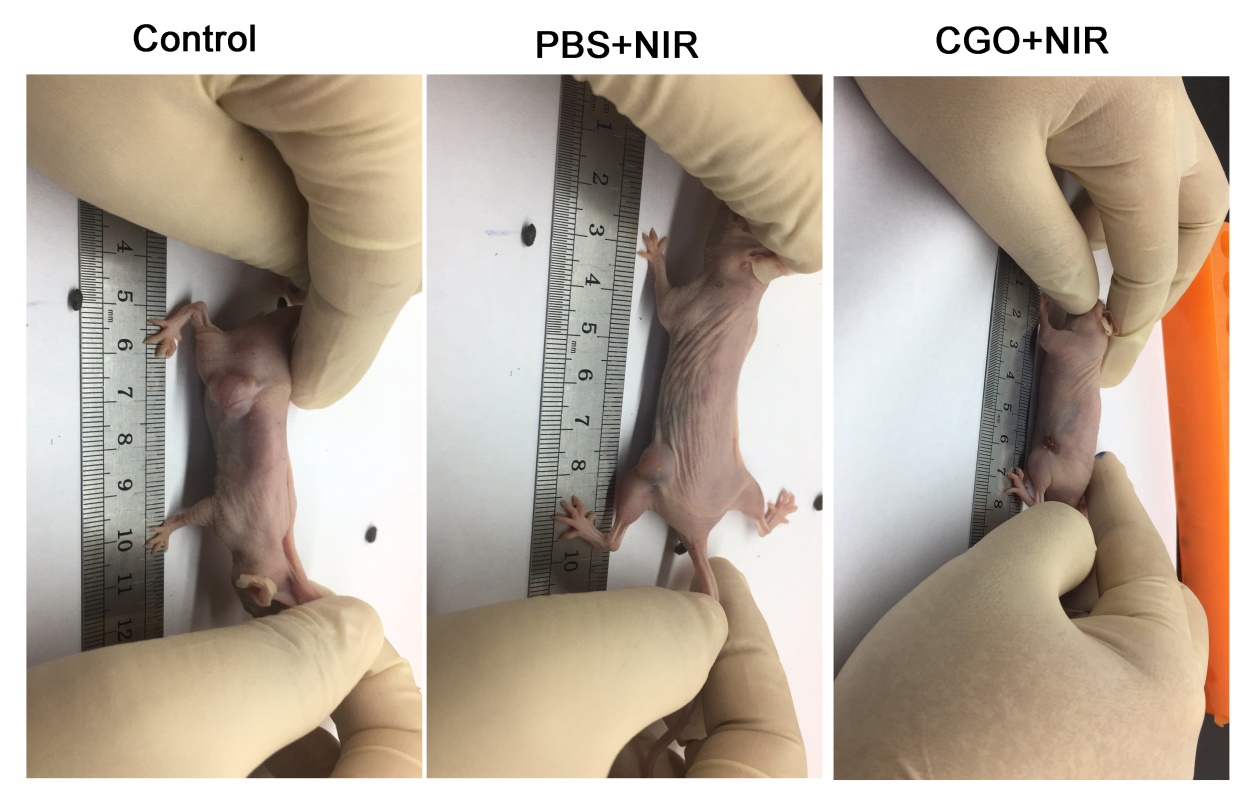


**Figure S2**. Pictures of mice in different groups after the indicated treatments at day 14^th^.


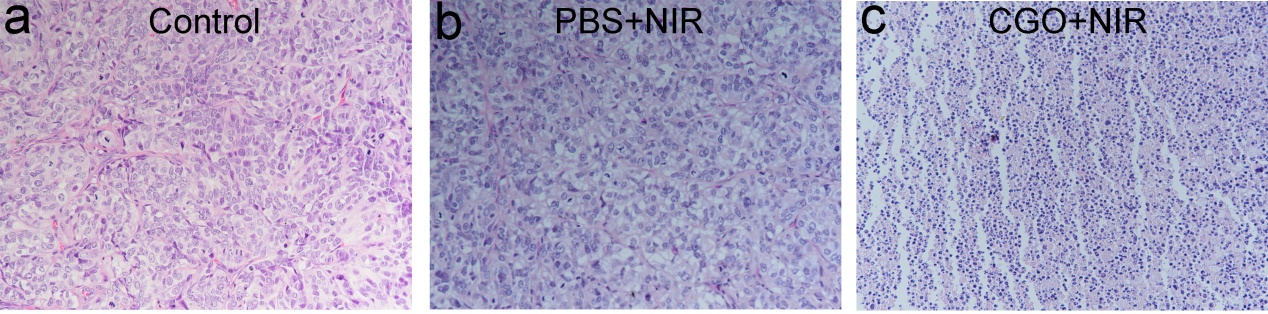


**Figure S3**. The representative hematoxylin and eosin (H&E) stained histological images of the corresponding ex vivo tumor sections: (a) control group, (b) PBS+NIR group, (c) CGO+NIR group. Magnification: 200 times.


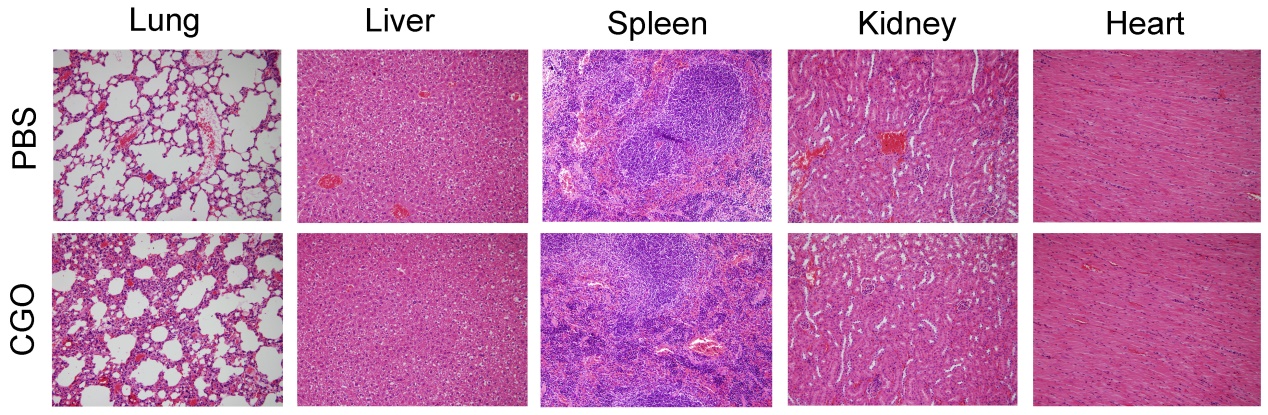


Figure S4. H&E stained slices of main organs. Magnification: 200 times.


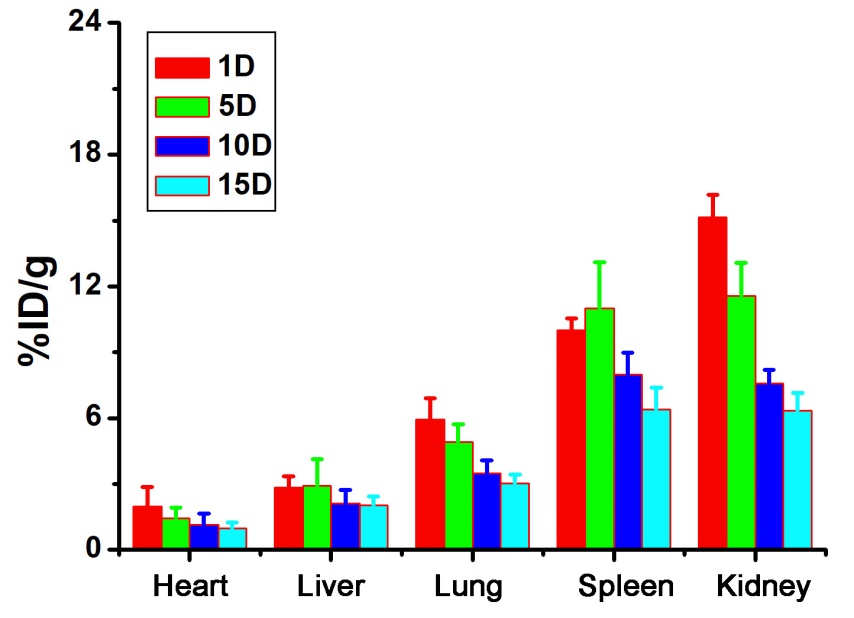


**Figure S5**. Biodistribution of CGO nanoparticles in major organs.
